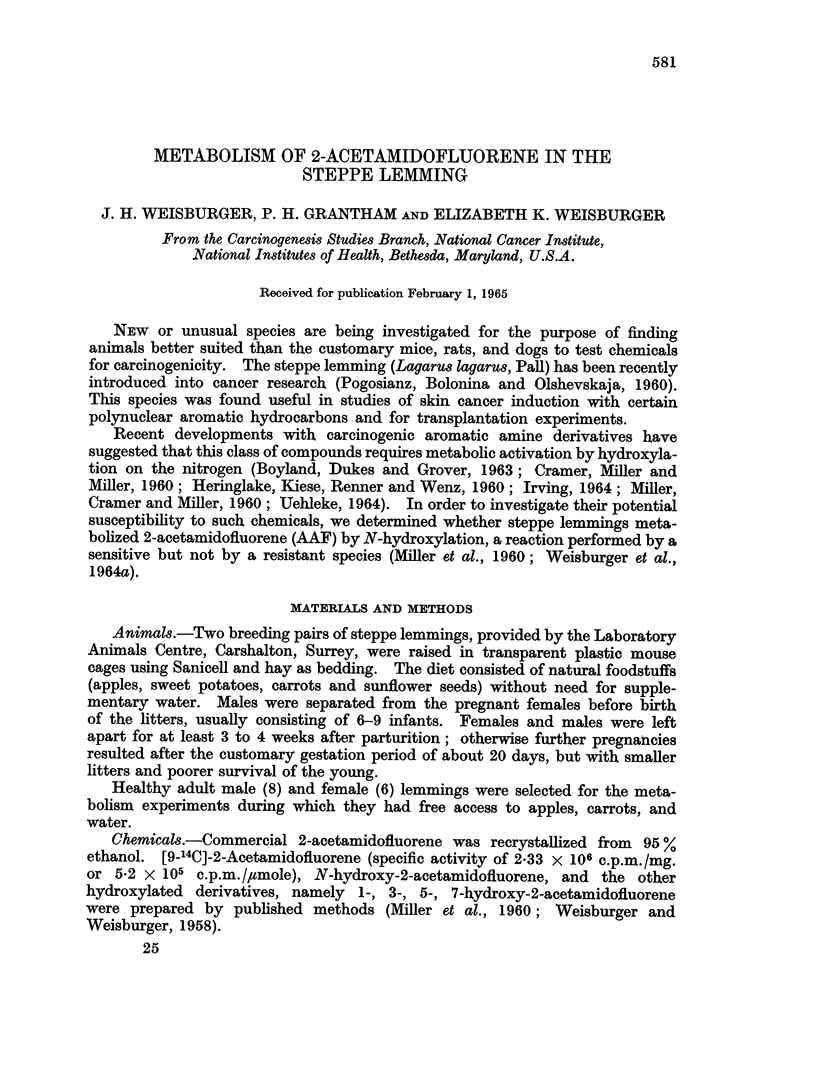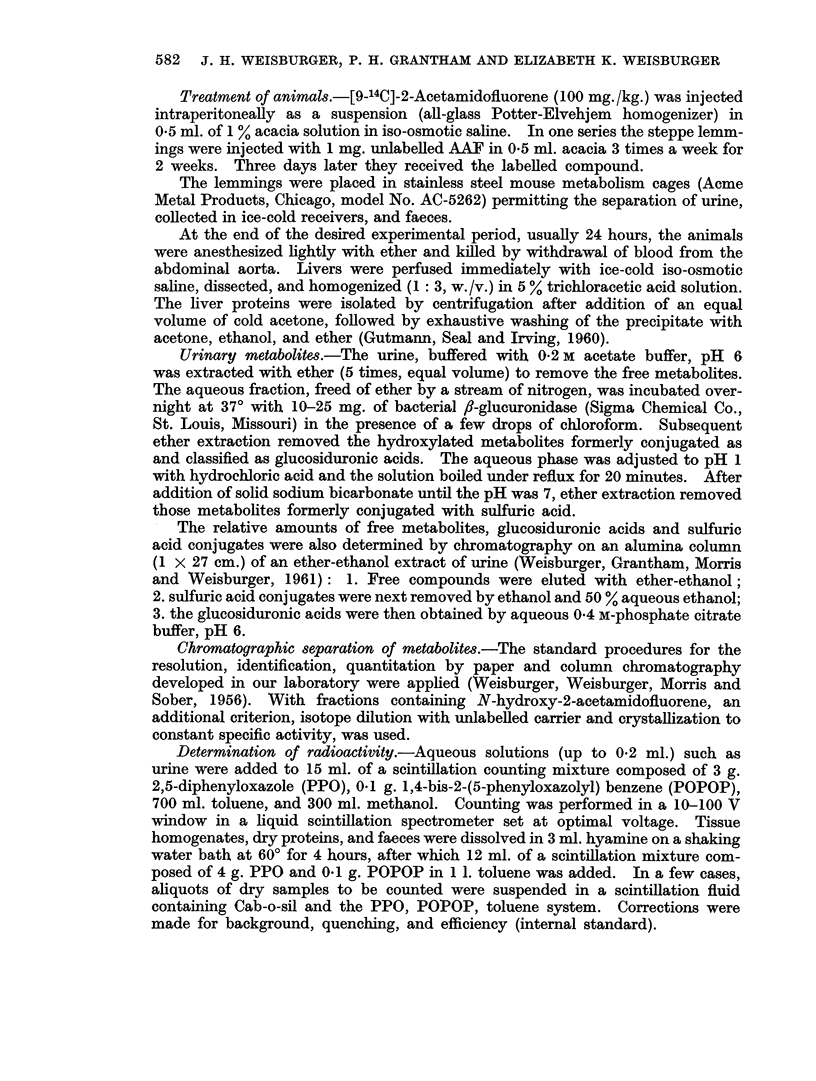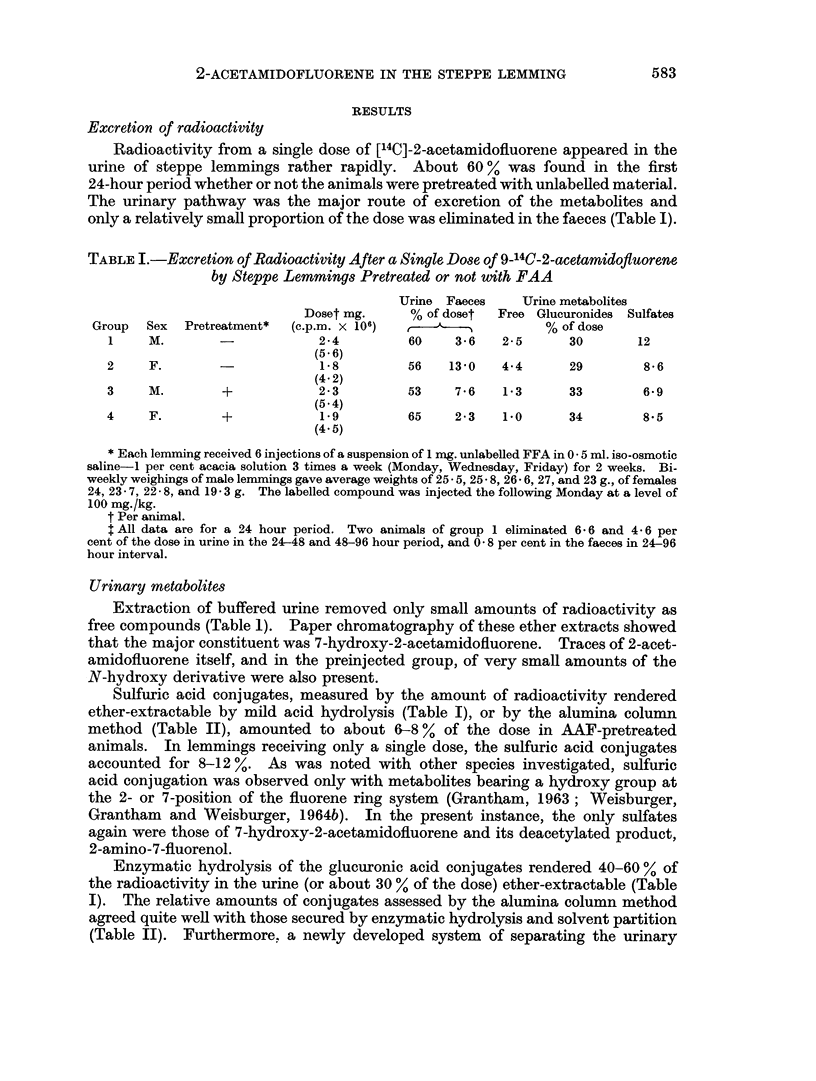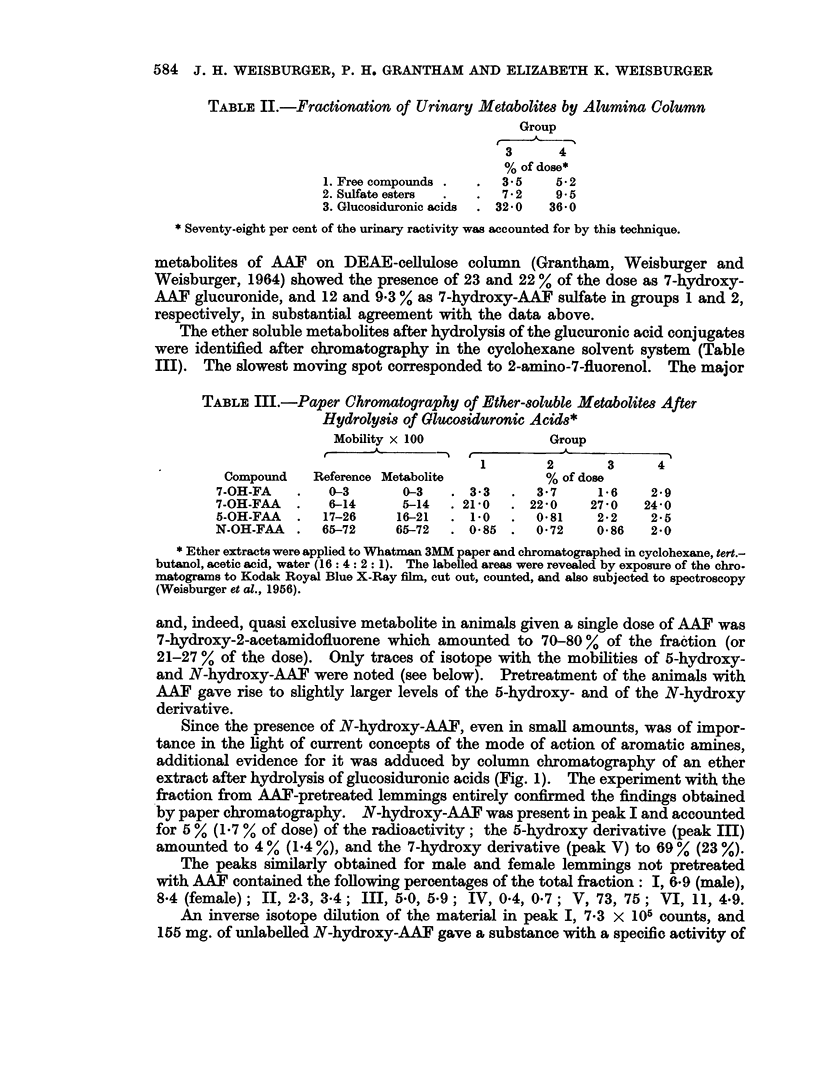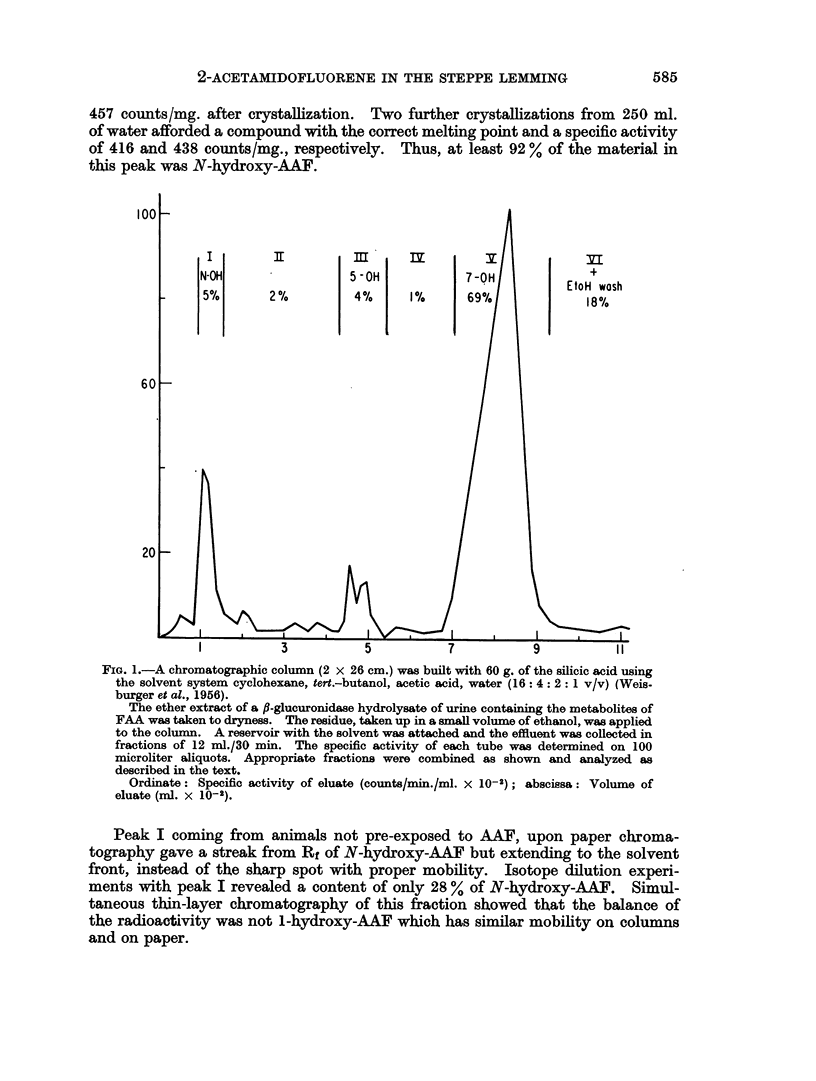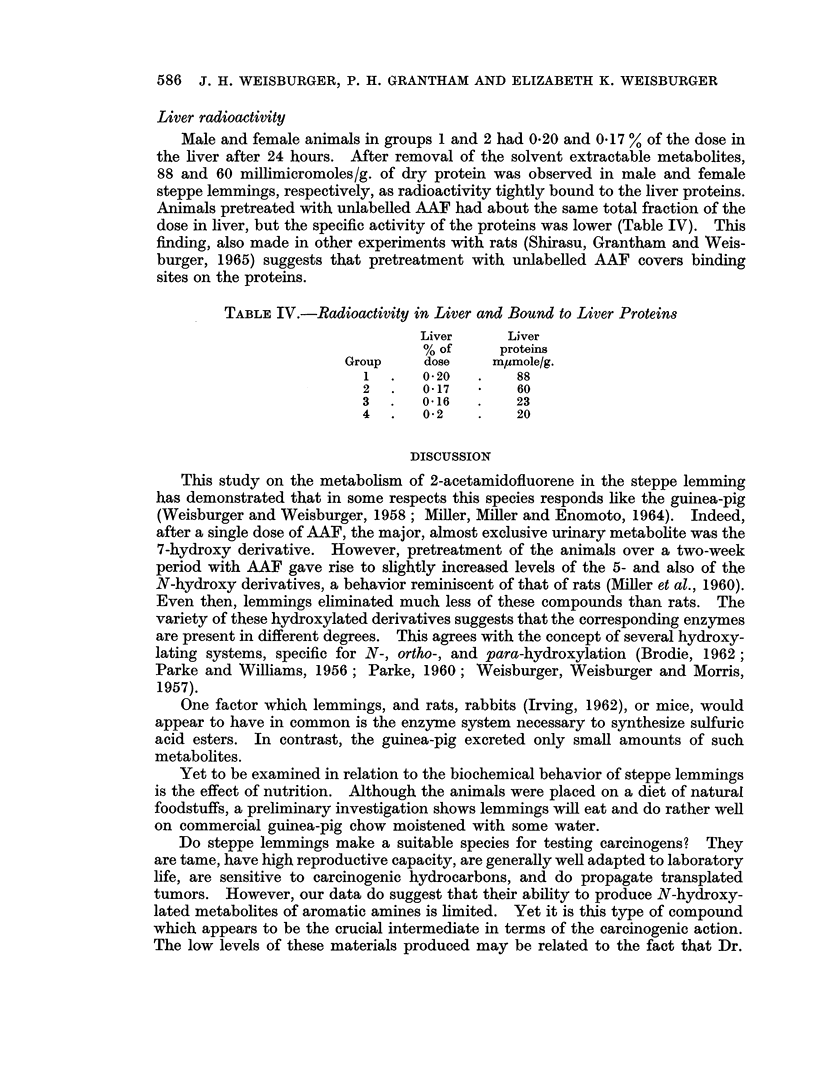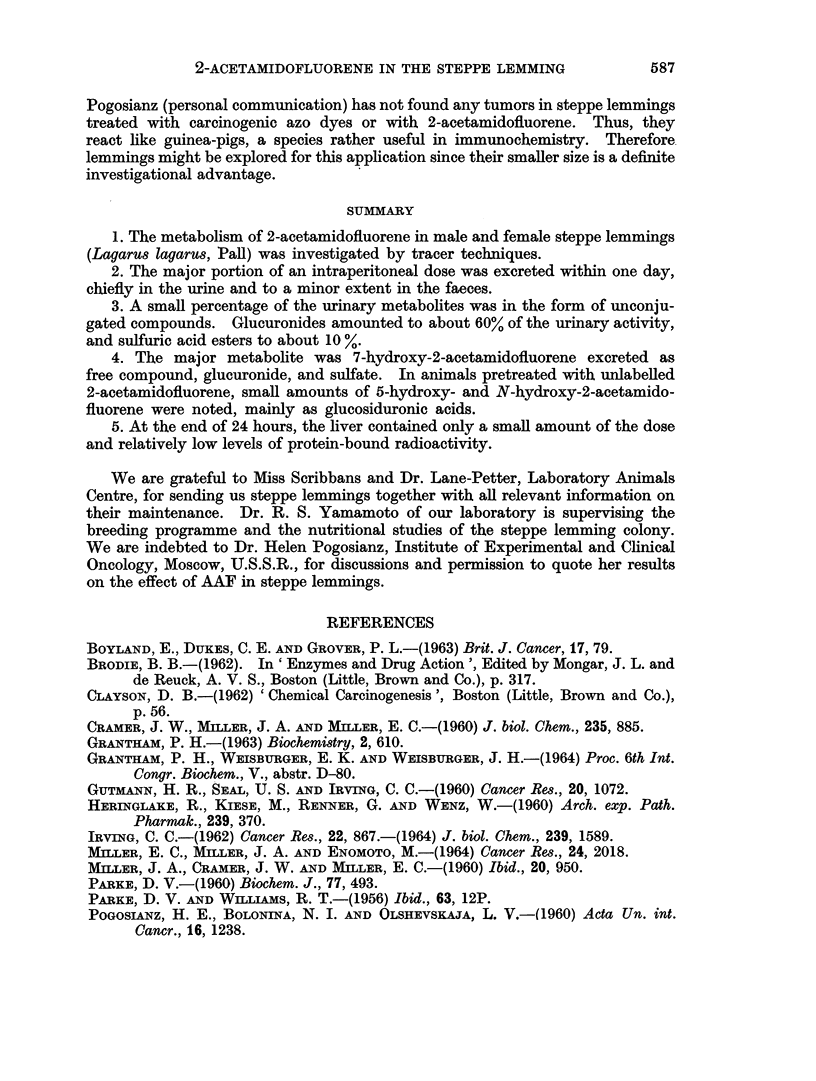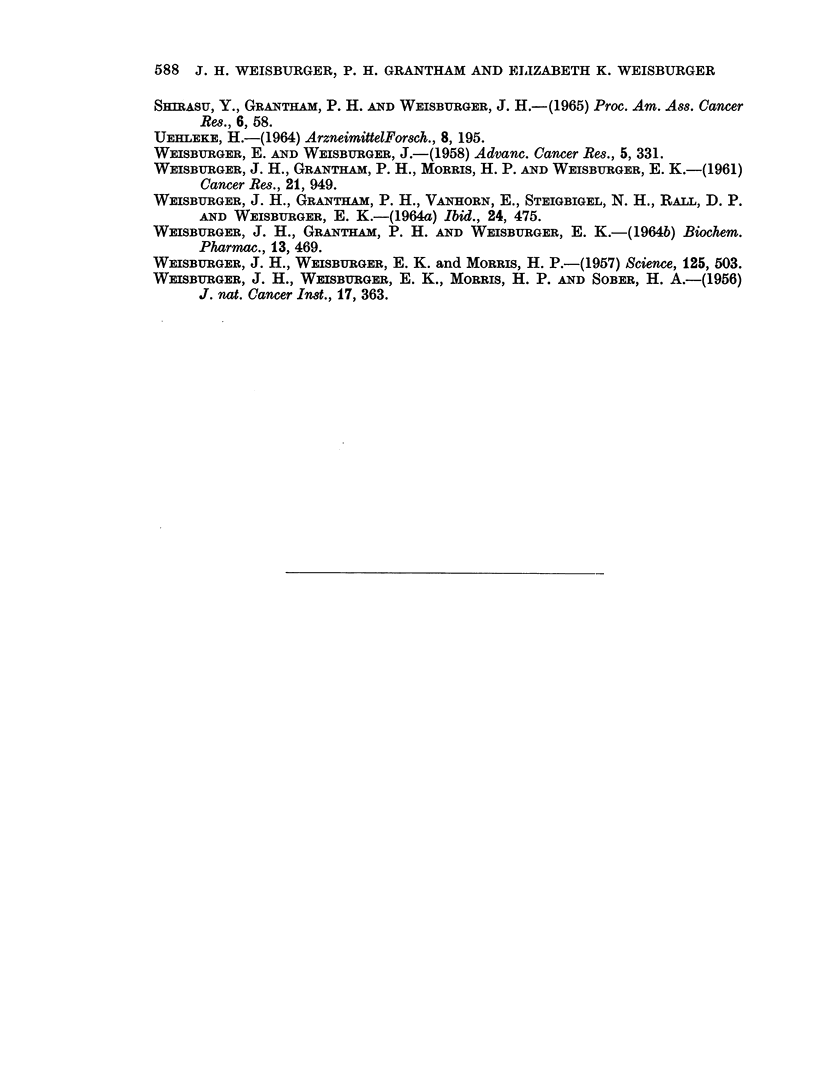# Metabolism of 2-acetamidofluorene in the steppe lemming.

**DOI:** 10.1038/bjc.1965.72

**Published:** 1965-09

**Authors:** J. H. Weisburger, P. H. Grantham, E. K. Weisburger


### METABOLISM OF 2-ACETAMIDOFLUORENE IN THE STEPPE LEMMING

J. H. WEISBURGER, P. H. GRANTHAM AND ELIZABETH K. WEISBURGER

*From the Carcinogenesis Studies Branch, National Cancer Institute, National Institutes of Health, Bethesda, Maryland, U.S.A.*

Received for publication February 1, 1965

NEW or unusual species are being investigated for the purpose of finding animals better suited than the customary mice, rats, and dogs to test chemicals for carcinogenicity. The steppe lemming (*Lagarus lagarus*, Pall) has been recently introduced into cancer research (Pogosianz, Bolonina and Olshevskaja, 1960). This species was found useful in studies of skin cancer induction with certain polynuclear aromatic hydrocarbons and for transplantation experiments.

Recent developments with carcinogenic aromatic amine derivatives have suggested that this class of compounds requires metabolic activation by hydroxylation on the nitrogen (Boyland, Dukes and Grover, 1963 ; Cramer, Miller and Miller, 1960 ; Heringlake, Kiese, Renner and Wenz, 1960 ; Irving, 1964 ; Miller, Cramer and Miller, 1960 ; Uehleke, 1964). In order to investigate their potential susceptibility to such chemicals, we determined whether steppe lemmings metabolized 2-acetamidofluorene (AAF) by *N*-hydroxylation, a reaction performed by a sensitive but not by a resistant species (Miller *et al.*, 1960 ; Weisburger *et al.*, 1964*a*).

### MATERIALS AND METHODS

*Animals.*—Two breeding pairs of steppe lemmings, provided by the Laboratory Animals Centre, Carshalton, Surrey, were raised in transparent plastic mouse cages using Sanicell and hay as bedding. The diet consisted of natural foodstuffs (apples, sweet potatoes, carrots and sunflower seeds) without need for supplementary water. Males were separated from the pregnant females before birth of the litters, usually consisting of 6–9 infants. Females and males were left apart for at least 3 to 4 weeks after parturition ; otherwise further pregnancies resulted after the customary gestation period of about 20 days, but with smaller litters and poorer survival of the young.

Healthy adult male (8) and female (6) lemmings were selected for the metabolism experiments during which they had free access to apples, carrots, and water.

*Chemicals.*—Commercial 2-acetamidofluorene was recrystallized from 95% ethanol. [9-$^{14}$C]-2-Acetamidofluorene (specific activity of $2{\cdot}33 \times 10^6$ c.p.m./mg. or $5{\cdot}2 \times 10^5$ c.p.m./$\mu$mole), *N*-hydroxy-2-acetamidofluorene, and the other hydroxylated derivatives, namely 1-, 3-, 5-, 7-hydroxy-2-acetamidofluorene were prepared by published methods (Miller *et al.*, 1960 ; Weisburger and Weisburger, 1958).

*Treatment of animals.*—[9-$^{14}$C]-2-Acetamidofluorene (100 mg./kg.) was injected intraperitoneally as a suspension (all-glass Potter-Elvehjem homogenizer) in 0·5 ml. of 1 % acacia solution in iso-osmotic saline. In one series the steppe lemmings were injected with 1 mg. unlabelled AAF in 0·5 ml. acacia 3 times a week for 2 weeks. Three days later they received the labelled compound.

The lemmings were placed in stainless steel mouse metabolism cages (Acme Metal Products, Chicago, model No. AC-5262) permitting the separation of urine, collected in ice-cold receivers, and faeces.

At the end of the desired experimental period, usually 24 hours, the animals were anesthesized lightly with ether and killed by withdrawal of blood from the abdominal aorta. Livers were perfused immediately with ice-cold iso-osmotic saline, dissected, and homogenized (1 : 3, w./v.) in 5 % trichloracetic acid solution. The liver proteins were isolated by centrifugation after addition of an equal volume of cold acetone, followed by exhaustive washing of the precipitate with acetone, ethanol, and ether (Gutmann, Seal and Irving, 1960).

*Urinary metabolites.*—The urine, buffered with 0·2 M acetate buffer, pH 6 was extracted with ether (5 times, equal volume) to remove the free metabolites. The aqueous fraction, freed of ether by a stream of nitrogen, was incubated overnight at 37° with 10–25 mg. of bacterial $\beta$-glucuronidase (Sigma Chemical Co., St. Louis, Missouri) in the presence of a few drops of chloroform. Subsequent ether extraction removed the hydroxylated metabolites formerly conjugated as and classified as glucosiduronic acids. The aqueous phase was adjusted to pH 1 with hydrochloric acid and the solution boiled under reflux for 20 minutes. After addition of solid sodium bicarbonate until the pH was 7, ether extraction removed those metabolites formerly conjugated with sulfuric acid.

The relative amounts of free metabolites, glucosiduronic acids and sulfuric acid conjugates were also determined by chromatography on an alumina column (1 × 27 cm.) of an ether-ethanol extract of urine (Weisburger, Grantham, Morris and Weisburger, 1961): 1. Free compounds were eluted with ether-ethanol; 2. sulfuric acid conjugates were next removed by ethanol and 50 % aqueous ethanol; 3. the glucosiduronic acids were then obtained by aqueous 0·4 M-phosphate citrate buffer, pH 6.

*Chromatographic separation of metabolites.*—The standard procedures for the resolution, identification, quantitation by paper and column chromatography developed in our laboratory were applied (Weisburger, Weisburger, Morris and Sober, 1956). With fractions containing *N*-hydroxy-2-acetamidofluorene, an additional criterion, isotope dilution with unlabelled carrier and crystallization to constant specific activity, was used.

*Determination of radioactivity.*—Aqueous solutions (up to 0·2 ml.) such as urine were added to 15 ml. of a scintillation counting mixture composed of 3 g. 2,5-diphenyloxazole (PPO), 0·1 g. 1,4-bis-2-(5-phenyloxazolyl) benzene (POPOP), 700 ml. toluene, and 300 ml. methanol. Counting was performed in a 10–100 V window in a liquid scintillation spectrometer set at optimal voltage. Tissue homogenates, dry proteins, and faeces were dissolved in 3 ml. hyamine on a shaking water bath at 60° for 4 hours, after which 12 ml. of a scintillation mixture composed of 4 g. PPO and 0·1 g. POPOP in 1 l. toluene was added. In a few cases, aliquots of dry samples to be counted were suspended in a scintillation fluid containing Cab-o-sil and the PPO, POPOP, toluene system. Corrections were made for background, quenching, and efficiency (internal standard).

### RESULTS

### *Excretion of radioactivity*

Radioactivity from a single dose of [$^{14}$C]-2-acetamidofluorene appeared in the urine of steppe lemmings rather rapidly. About 60% was found in the first 24-hour period whether or not the animals were pretreated with unlabelled material. The urinary pathway was the major route of excretion of the metabolites and only a relatively small proportion of the dose was eliminated in the faeces (Table I).

TABLE I.—*Excretion of Radioactivity After a Single Dose of* 9-$^{14}$*C*-2-*acetamidofluorene by Steppe Lemmings Pretreated or not with FAA*

| Group | Sex | Pretreatment* | Dose† mg. (c.p.m. × $10^6$) | Urine (% of dose†) | Faeces (% of dose†) | Urine metabolites: Free (% of dose) | Glucuronides (% of dose) | Sulfates (% of dose) |
|---|---|---|---|---|---|---|---|---|
| 1 | M. | — | 2·4 (5·6) | 60 | 3·6 | 2·5 | 30 | 12 |
| 2 | F. | — | 1·8 (4·2) | 56 | 13·0 | 4·4 | 29 | 8·6 |
| 3 | M. | + | 2·3 (5·4) | 53 | 7·6 | 1·3 | 33 | 6·9 |
| 4 | F. | + | 1·9 (4·5) | 65 | 2·3 | 1·0 | 34 | 8·5 |

* Each lemming received 6 injections of a suspension of 1 mg. unlabelled FFA in 0·5 ml. iso-osmotic saline—1 per cent acacia solution 3 times a week (Monday, Wednesday, Friday) for 2 weeks. Biweekly weighings of male lemmings gave average weights of 25·5, 25·8, 26·6, 27, and 23 g., of females 24, 23·7, 22·8, and 19·3 g. The labelled compound was injected the following Monday at a level of 100 mg./kg.

† Per animal.

‡ All data are for a 24 hour period. Two animals of group 1 eliminated 6·6 and 4·6 per cent of the dose in urine in the 24–48 and 48–96 hour period, and 0·8 per cent in the faeces in 24–96 hour interval.

### *Urinary metabolites*

Extraction of buffered urine removed only small amounts of radioactivity as free compounds (Table 1). Paper chromatography of these ether extracts showed that the major constituent was 7-hydroxy-2-acetamidofluorene. Traces of 2-acetamidofluorene itself, and in the preinjected group, of very small amounts of the *N*-hydroxy derivative were also present.

Sulfuric acid conjugates, measured by the amount of radioactivity rendered ether-extractable by mild acid hydrolysis (Table I), or by the alumina column method (Table II), amounted to about 6–8% of the dose in AAF-pretreated animals. In lemmings receiving only a single dose, the sulfuric acid conjugates accounted for 8–12%. As was noted with other species investigated, sulfuric acid conjugation was observed only with metabolites bearing a hydroxy group at the 2- or 7-position of the fluorene ring system (Grantham, 1963; Weisburger, Grantham and Weisburger, 1964*b*). In the present instance, the only sulfates again were those of 7-hydroxy-2-acetamidofluorene and its deacetylated product, 2-amino-7-fluorenol.

Enzymatic hydrolysis of the glucuronic acid conjugates rendered 40–60% of the radioactivity in the urine (or about 30% of the dose) ether-extractable (Table I). The relative amounts of conjugates assessed by the alumina column method agreed quite well with those secured by enzymatic hydrolysis and solvent partition (Table II). Furthermore, a newly developed system of separating the urinary

TABLE II.—*Fractionation of Urinary Metabolites by Alumina Column*

| | Group 3 | Group 4 |
|---|---|---|
| | % of dose* | |
| 1. Free compounds . . | 3·5 | 5·2 |
| 2. Sulfate esters . . | 7·2 | 9·5 |
| 3. Glucosiduronic acids . | 32·0 | 36·0 |

* Seventy-eight per cent of the urinary ractivity was accounted for by this technique.

metabolites of AAF on DEAE-cellulose column (Grantham, Weisburger and Weisburger, 1964) showed the presence of 23 and 22 % of the dose as 7-hydroxy-AAF glucuronide, and 12 and 9·3 % as 7-hydroxy-AAF sulfate in groups 1 and 2, respectively, in substantial agreement with the data above.

The ether soluble metabolites after hydrolysis of the glucuronic acid conjugates were identified after chromatography in the cyclohexane solvent system (Table III). The slowest moving spot corresponded to 2-amino-7-fluorenol. The major

TABLE III.—*Paper Chromatography of Ether-soluble Metabolites After Hydrolysis of Glucosiduronic Acids**

| | Mobility × 100 | | Group | | | |
|---|---|---|---|---|---|---|
| | | | 1 | 2 | 3 | 4 |
| Compound | Reference | Metabolite | | % of dose | | |
| 7-OH-FA . | 0–3 | 0–3 . | 3·3 . | 3·7 | 1·6 | 2·9 |
| 7-OH-FAA . | 6–14 | 5–14 . | 21·0 . | 22·0 | 27·0 | 24·0 |
| 5-OH-FAA . | 17–26 | 16–21 . | 1·0 . | 0·81 | 2·2 | 2·5 |
| N-OH-FAA . | 65–72 | 65–72 . | 0·85 . | 0·72 | 0·86 | 2·0 |

* Ether extracts were applied to Whatman 3MM paper and chromatographed in cyclohexane, *tert.*–butanol, acetic acid, water (16 : 4 : 2 : 1). The labelled areas were revealed by exposure of the chromatograms to Kodak Royal Blue X-Ray film, cut out, counted, and also subjected to spectroscopy (Weisburger *et al.*, 1956).

and, indeed, quasi exclusive metabolite in animals given a single dose of AAF was 7-hydroxy-2-acetamidofluorene which amounted to 70–80 % of the fraction (or 21–27 % of the dose). Only traces of isotope with the mobilities of 5-hydroxy- and *N*-hydroxy-AAF were noted (see below). Pretreatment of the animals with AAF gave rise to slightly larger levels of the 5-hydroxy- and of the *N*-hydroxy derivative.

Since the presence of *N*-hydroxy-AAF, even in small amounts, was of importance in the light of current concepts of the mode of action of aromatic amines, additional evidence for it was adduced by column chromatography of an ether extract after hydrolysis of glucosiduronic acids (Fig. 1). The experiment with the fraction from AAF-pretreated lemmings entirely confirmed the findings obtained by paper chromatography. *N*-hydroxy-AAF was present in peak I and accounted for 5 % (1·7 % of dose) of the radioactivity ; the 5-hydroxy derivative (peak III) amounted to 4 % (1·4 %), and the 7-hydroxy derivative (peak V) to 69 % (23 %).

The peaks similarly obtained for male and female lemmings not pretreated with AAF contained the following percentages of the total fraction : I, 6·9 (male), 8·4 (female) ; II, 2·3, 3·4 ; III, 5·0, 5·9 ; IV, 0·4, 0·7 ; V, 73, 75 ; VI, 11, 4·9.

An inverse isotope dilution of the material in peak I, 7·3 × $10^5$ counts, and 155 mg. of unlabelled *N*-hydroxy-AAF gave a substance with a specific activity of

457 counts/mg. after crystallization. Two further crystallizations from 250 ml. of water afforded a compound with the correct melting point and a specific activity of 416 and 438 counts/mg., respectively. Thus, at least 92 % of the material in this peak was *N*-hydroxy-AAF.

FIG. 1.—A chromatographic column (2 × 26 cm.) was built with 60 g. of the silicic acid using the solvent system cyclohexane, *tert.*–butanol, acetic acid, water (16 : 4 : 2 : 1 v/v) (Weisburger *et al.*, 1956).

The ether extract of a $\beta$-glucuronidase hydrolysate of urine containing the metabolites of FAA was taken to dryness. The residue, taken up in a small volume of ethanol, was applied to the column. A reservoir with the solvent was attached and the effluent was collected in fractions of 12 ml./30 min. The specific activity of each tube was determined on 100 microliter aliquots. Appropriate fractions were combined as shown and analyzed as described in the text.

Ordinate: Specific activity of eluate (counts/min./ml. × $10^{-2}$); abscissa: Volume of eluate (ml. × $10^{-2}$).

Peak I coming from animals not pre-exposed to AAF, upon paper chromatography gave a streak from $R_f$ of *N*-hydroxy-AAF but extending to the solvent front, instead of the sharp spot with proper mobility. Isotope dilution experiments with peak I revealed a content of only 28 % of *N*-hydroxy-AAF. Simultaneous thin-layer chromatography of this fraction showed that the balance of the radioactivity was not 1-hydroxy-AAF which has similar mobility on columns and on paper.

*Liver radioactivity*

Male and female animals in groups 1 and 2 had 0·20 and 0·17 % of the dose in the liver after 24 hours. After removal of the solvent extractable metabolites, 88 and 60 millimicromoles/g. of dry protein was observed in male and female steppe lemmings, respectively, as radioactivity tightly bound to the liver proteins. Animals pretreated with unlabelled AAF had about the same total fraction of the dose in liver, but the specific activity of the proteins was lower (Table IV). This finding, also made in other experiments with rats (Shirasu, Grantham and Weisburger, 1965) suggests that pretreatment with unlabelled AAF covers binding sites on the proteins.

TABLE IV.—*Radioactivity in Liver and Bound to Liver Proteins*

| Group | Liver % of dose | Liver proteins mμmole/g. |
|---|---|---|
| 1 | 0·20 | 88 |
| 2 | 0·17 | 60 |
| 3 | 0·16 | 23 |
| 4 | 0·2 | 20 |

### DISCUSSION

This study on the metabolism of 2-acetamidofluorene in the steppe lemming has demonstrated that in some respects this species responds like the guinea-pig (Weisburger and Weisburger, 1958 ; Miller, Miller and Enomoto, 1964). Indeed, after a single dose of AAF, the major, almost exclusive urinary metabolite was the 7-hydroxy derivative. However, pretreatment of the animals over a two-week period with AAF gave rise to slightly increased levels of the 5- and also of the *N*-hydroxy derivatives, a behavior reminiscent of that of rats (Miller *et al.*, 1960). Even then, lemmings eliminated much less of these compounds than rats. The variety of these hydroxylated derivatives suggests that the corresponding enzymes are present in different degrees. This agrees with the concept of several hydroxylating systems, specific for *N*-, *ortho*-, and *para*-hydroxylation (Brodie, 1962 ; Parke and Williams, 1956 ; Parke, 1960 ; Weisburger, Weisburger and Morris, 1957).

One factor which lemmings, and rats, rabbits (Irving, 1962), or mice, would appear to have in common is the enzyme system necessary to synthesize sulfuric acid esters. In contrast, the guinea-pig excreted only small amounts of such metabolites.

Yet to be examined in relation to the biochemical behavior of steppe lemmings is the effect of nutrition. Although the animals were placed on a diet of natural foodstuffs, a preliminary investigation shows lemmings will eat and do rather well on commercial guinea-pig chow moistened with some water.

Do steppe lemmings make a suitable species for testing carcinogens? They are tame, have high reproductive capacity, are generally well adapted to laboratory life, are sensitive to carcinogenic hydrocarbons, and do propagate transplated tumors. However, our data do suggest that their ability to produce *N*-hydroxylated metabolites of aromatic amines is limited. Yet it is this type of compound which appears to be the crucial intermediate in terms of the carcinogenic action. The low levels of these materials produced may be related to the fact that Dr.

Pogosianz (personal communication) has not found any tumors in steppe lemmings treated with carcinogenic azo dyes or with 2-acetamidofluorene. Thus, they react like guinea-pigs, a species rather useful in immunochemistry. Therefore lemmings might be explored for this application since their smaller size is a definite investigational advantage.

### SUMMARY

1. The metabolism of 2-acetamidofluorene in male and female steppe lemmings (*Lagarus lagarus*, Pall) was investigated by tracer techniques.

2. The major portion of an intraperitoneal dose was excreted within one day, chiefly in the urine and to a minor extent in the faeces.

3. A small percentage of the urinary metabolites was in the form of unconjugated compounds. Glucuronides amounted to about 60% of the urinary activity, and sulfuric acid esters to about 10 %.

4. The major metabolite was 7-hydroxy-2-acetamidofluorene excreted as free compound, glucuronide, and sulfate. In animals pretreated with unlabelled 2-acetamidofluorene, small amounts of 5-hydroxy- and *N*-hydroxy-2-acetamidofluorene were noted, mainly as glucosiduronic acids.

5. At the end of 24 hours, the liver contained only a small amount of the dose and relatively low levels of protein-bound radioactivity.

We are grateful to Miss Scribbans and Dr. Lane-Petter, Laboratory Animals Centre, for sending us steppe lemmings together with all relevant information on their maintenance. Dr. R. S. Yamamoto of our laboratory is supervising the breeding programme and the nutritional studies of the steppe lemming colony. We are indebted to Dr. Helen Pogosianz, Institute of Experimental and Clinical Oncology, Moscow, U.S.S.R., for discussions and permission to quote her results on the effect of AAF in steppe lemmings.

### REFERENCES

BOYLAND, E., DUKES, C. E. AND GROVER, P. L.—(1963) *Brit. J. Cancer*, **17**, 79.

BRODIE, B. B.—(1962). In ‘ Enzymes and Drug Action ’, Edited by Mongar, J. L. and de Reuck, A. V. S., Boston (Little, Brown and Co.), p. 317.

CLAYSON, D. B.—(1962) ‘ Chemical Carcinogenesis ’, Boston (Little, Brown and Co.), p. 56.

CRAMER, J. W., MILLER, J. A. AND MILLER, E. C.—(1960) *J. biol. Chem.*, **235**, 885.

GRANTHAM, P. H.—(1963) *Biochemistry*, **2**, 610.

GRANTHAM, P. H., WEISBURGER, E. K. AND WEISBURGER, J. H.—(1964) *Proc. 6th Int. Congr. Biochem.*, V., abstr. D–80.

GUTMANN, H. R., SEAL, U. S. AND IRVING, C. C.—(1960) *Cancer Res.*, **20**, 1072.

HERINGLAKE, R., KIESE, M., RENNER, G. AND WENZ, W.—(1960) *Arch. exp. Path. Pharmak.*, **239**, 370.

IRVING, C. C.—(1962) *Cancer Res.*, **22**, 867.—(1964) *J. biol. Chem.*, **239**, 1589.

MILLER, E. C., MILLER, J. A. AND ENOMOTO, M.—(1964) *Cancer Res.*, **24**, 2018.

MILLER, J. A., CRAMER, J. W. AND MILLER, E. C.—(1960) *Ibid.*, **20**, 950.

PARKE, D. V.—(1960) *Biochem. J.*, **77**, 493.

PARKE, D. V. AND WILLIAMS, R. T.—(1956) *Ibid.*, **63**, 12P.

POGOSIANZ, H. E., BOLONINA, N. I. AND OLSHEVSKAJA, L. V.—(1960) *Acta Un. int. Cancr.*, **16**, 1238.

Shirasu, Y., Grantham, P. H. and Weisburger, J. H.—(1965) *Proc. Am. Ass. Cancer Res.*, **6**, 58.

Uehleke, H.—(1964) *ArzneimittelForsch.*, **8**, 195.

Weisburger, E. and Weisburger, J.—(1958) *Advanc. Cancer Res.*, **5**, 331.

Weisburger, J. H., Grantham, P. H., Morris, H. P. and Weisburger, E. K.—(1961) *Cancer Res.*, **21**, 949.

Weisburger, J. H., Grantham, P. H., Vanhorn, E., Steigbigel, N. H., Rall, D. P. and Weisburger, E. K.—(1964*a*) *Ibid.*, **24**, 475.

Weisburger, J. H., Grantham, P. H. and Weisburger, E. K.—(1964*b*) *Biochem. Pharmac.*, **13**, 469.

Weisburger, J. H., Weisburger, E. K. and Morris, H. P.—(1957) *Science*, **125**, 503.

Weisburger, J. H., Weisburger, E. K., Morris, H. P. and Sober, H. A.—(1956) *J. nat. Cancer Inst.*, **17**, 363.